# Fuzzy Information Discrimination Measures and Their Application to Low Dimensional Embedding Construction in the UMAP Algorithm

**DOI:** 10.3390/jimaging8040113

**Published:** 2022-04-15

**Authors:** Liliya A. Demidova, Artyom V. Gorchakov

**Affiliations:** Institute of Information Technologies, Federal State Budget Educational Institution of Higher Education “MIREA–Russian Technological University”, 78, Vernadsky Avenue, 119454 Moscow, Russia

**Keywords:** dimension reduction, data visualization, entropy, cross-entropy, fuzzy logic

## Abstract

Dimensionality reduction techniques are often used by researchers in order to make high dimensional data easier to interpret visually, as data visualization is only possible in low dimensional spaces. Recent research in nonlinear dimensionality reduction introduced many effective algorithms, including t-distributed stochastic neighbor embedding (t-SNE), uniform manifold approximation and projection (UMAP), dimensionality reduction technique based on triplet constraints (TriMAP), and pairwise controlled manifold approximation (PaCMAP), aimed to preserve both the local and global structure of high dimensional data while reducing the dimensionality. The UMAP algorithm has found its application in bioinformatics, genetics, genomics, and has been widely used to improve the accuracy of other machine learning algorithms. In this research, we compare the performance of different fuzzy information discrimination measures used as loss functions in the UMAP algorithm while constructing low dimensional embeddings. In order to achieve this, we derive the gradients of the considered losses analytically and employ the Adam algorithm during the loss function optimization process. From the conducted experimental studies we conclude that the use of either the logarithmic fuzzy cross entropy loss without reduced repulsion or the symmetric logarithmic fuzzy cross entropy loss with sufficiently large neighbor count leads to better global structure preservation of the original multidimensional data when compared to the loss function used in the original UMAP algorithm implementation.

## 1. Introduction

Research in artificial intelligence and machine learning introduced plenty of algorithms that are now widely used in the automation of processes that earlier required human intervention. Such algorithms include neural networks [1], extreme learning machines [2], support vector machines [3,4], and other algorithms that are often used by researchers and practitioners in order to solve classification, regression and clustering problems. These algorithms often work with objects represented by high dimensional vectors, and high dimensional data, as well as the decisions made by a trained machine learning algorithm, which might be hard or barely possible to interpret.

Dimension reduction algorithms address the described problem by making high dimensional data visually interpretable. A typical dimensionality reduction algorithm accepts a dataset with objects represented as high dimensional vectors, and outputs a new dataset, containing low dimensional vectors representing the same objects from the original dataset. Data visualization is only possible in two- or three-dimensional spaces. Hence, if a dimensionality reduction algorithm reduces the number of components in vectors representing objects from the original dataset to either two or three, then one will be able to easily visualize the dataset as a scatter plot.

Dimensionality reduction methods are commonly divided into linear and nonlinear approaches [5]. An example of a linear dimensionality reduction algorithm is Principal Component Analysis (PCA) [6], which seeks a linear projection of data to low dimensional space maximizing the variance. Nonlinear dimensionality reduction methods include Sammon’s mapping [7], Laplacian eigenmaps [8], t-distributed Stochastic neighbor embedding (t-SNE) [9], Uniform Manifold Approximation and Projection (UMAP) [10], dimensionality reduction technique based on triplet constraints (TriMAP) [11] and others. Both t-SNE and UMAP are widely used effective nonlinear manifold learning techniques that construct a weighted graph representing pairwise object similarities, and then embed high dimensional objects into low dimensional space based on the weighted graph.

Since the first mention of UMAP in [10], the algorithm has been applied to many different domains, including physical and genetic interactions visualization [12], single-cell data visualization [13], and spatio-temporal hydrological gridded datasets visualization [14]. Except for high dimensional data visualization, known UMAP applications include the improvement of different clustering algorithms by reducing the dimensionality of the original dataset [15,16]. In [16], a UMAP-assisted K-means algorithm was used to solve the clustering problem of large-scale SARS-CoV-2 mutation datasets, and the hybrid UMAP-based algorithm showed superior clustering accuracy and performance. In addition, the authors compared the visualizations of the datasets obtained after performing dimensionality reduction with PCA, t-SNE, and UMAP, and the latter algorithm managed to maintain more of the global structure of the data. In [17], UMAP was used in conjunction with the hierarchical density-based spatial clustering of applications with noise (HDBSCAN) algorithm, and this allowed for the significant enhancement of the silhouette score in time series clustering. In [18], a new semi-supervised approach based on UMAP was introduced and applied to minimal residual disease quantification.

UMAP has the potential to preserve more of the global structure of a high dimensional dataset after performing dimensionality reduction when compared to t-SNE [10]. The TriMAP algorithm can preserve global structure even better [11], but according to [16] the algorithm sometimes struggles with local structure preservation. In [19], a novel algorithm named PaCMAP was proposed as a result of a comprehensive comparative study of t-SNE, UMAP, and TriMAP. The authors of [19] show that the choice of loss function drastically affects the performance of a nonlinear manifold learning algorithm.

According to [10], the traditional UMAP algorithm uses fuzzy cross entropy [20,21] as a loss function. The reference implementation of the considered dimensionality reduction algorithm incorporates a sampling-based approach while performing gradient descent for the sake of performance, and this allows UMAP to process large datasets at a reasonable time. However, this feature also makes the incorporation of custom losses into the considered dimensionality reduction algorithm overly complex. As a result, loss functions other than the sampling-based fuzzy cross entropy that is described in [10] are yet to be studied. According to [19], the choice of a loss function greatly influences a manifold learning algorithm’s performance, so the incorporation of different loss functions other than the default one that is described in [10] could possibly lead to different, and potentially improved, low dimensional embeddings and their visualizations.

Recent research by [22] shows that the incorporation of a sampling-based approach while performing gradient descent leads to the weight constants of the loss used in the original UMAP implementation [10] being a bit different when compared to the well-known fuzzy cross entropy loss defined in [20,21]. In [22], the authors experimentally prove that UMAP significantly reduces repulsive weight in the original fuzzy cross entropy formula. The authors of [22] derive the true loss function formula that is used in the UMAP algorithm. Aside from the weighted fuzzy cross entropy with reduced repulsion that is actually used in the original UMAP algorithm, different measures of information discrimination between two fuzzy sets exist. Such measures include the original logarithmic fuzzy cross entropy that is based on Shannon entropy [23], symmetric fuzzy cross entropy [21], and modified fuzzy cross entropy [23,24].

In this research, we reimplement the UMAP algorithm from scratch without using the sampling-based approach during the loss function optimization process. This allows us to incorporate custom loss functions into the UMAP algorithm, and to investigate the performance of different fuzzy information discrimination measures optimized during low dimensional embedding construction that is performed by the UMAP algorithm. We employ the state-of-the-art Adam algorithm [25] during the optimization process. The Adam algorithm is a first-order optimization method. First-order optimization methods exploit information on values and gradients of an optimized function. Hence, we have to derive the gradients of the considered loss functions analytically. After deriving the gradients of the losses, we compare the visualizations obtained while using different losses with different UMAP hyperparameters.

Based on the findings described in [19], the use of loss functions other than the default sampling-based one [10] could possibly lead to different low dimensional embeddings, that potentially better preserve the original structure of a multidimensional dataset. This might simplify the visual interpretability of the data in different domains [12,14], as well as positively affect the accuracy of clustering algorithms based on the preliminary evaluation of the UMAP algorithm.

The results of the study show that the use of either the original logarithmic fuzzy cross entropy or symmetric fuzzy cross entropy leads to better global structure preservation of the original dataset, in case the nearest neighbor count is sufficiently large.

## 2. Materials and Methods

### 2.1. Fuzzy Weighted Undirected Graph Construction in the UMAP Algorithm

The UMAP algorithm has the potential to better preserve both the local and global structure of high dimensional data while performing nonlinear dimensionality reduction, when compared to algorithms such as PCA, multidimensional scaling (MDS), t-SNE, and LargeVis [10].

Recent findings show that the original UMAP implementation optimizes fuzzy cross entropy with drastically reduced repulsion [22], but not the original fuzzy cross entropy as defined in [20,21]. According to [19], the choice of loss function drastically affects the performance of a nonlinear manifold learning algorithm. The reference implementation of the UMAP algorithm uses a sampling-based approach for the sake of performance [10], and this complicates the extensibility of the UMAP with custom losses. Therefore, we reimplement the UMAP algorithm from scratch with an intention to investigate the performance of the considered nonlinear dimensionality reduction technique with different fuzzy information discrimination measures [21,23,24] used as loss functions while constructing low dimensional embeddings.

In this section, we briefly describe the considered manifold learning algorithm. The UMAP algorithm consists of two phases, a fuzzy weighted undirected graph is constructed during the first phase of the nonlinear dimensionality reduction process, and the loss function is optimized during the second phase.

The UMAP algorithm accepts a dataset $X=\left\{{\vec{x}}_{1},{\vec{x}}_{2},\ldots ,{\vec{x}}_{n}\right\}$, which contains n objects. Every object ${\vec{x}}_{i}\in X$ is represented by an h-dimensional vector containing real numbers. In order words, $\forall {\vec{x}}_{i}\in X:{\vec{x}}_{i}\in {\mathbb{R}}^{h}$. First, the algorithm searches for k nearest neighbors $T_{i}=\left\{{\vec{t}}_{i1},\ldots ,{\vec{t}}_{il},\ldots ,{\vec{t}}_{ik}\right\}$ for every object ${\vec{x}}_{i}\in X$, assuming $\forall {\vec{t}}_{il}\in T_{i}:{\vec{t}}_{il}\in X$. The k nearest neighbor search is performed using the approach proposed in [26]. For every found neighbor from the $T_{i}$ set, the scalar distance value $d_{il}$ between ${\vec{x}}_{i}$ and ${\vec{t}}_{il}\in T_{i}$ is computed using a distance metric. The distance metric used for this step is the hyperparameter of the UMAP algorithm. In the case that one uses the Euclidean distance metric, the scalar $d_{il}$ value is computed as follows:

(1) $$d_{il}=\left|⏐{\vec{x}}_{i}-{\vec{t}}_{il}⏐\right|=\sqrt{{\left(x_{i,1}-t_{il,1}\right)}^{2}+\cdots +{\left(x_{i,h}-t_{il,h}\right)}^{2}},$$

where i is the number of an object from the X set; l is the number of one of the k nearest neighbors of the *i*-th object; h denotes the dimensionality of the ${\vec{x}}_{i}\in X$ vector representing the *i*-th object, the dimensionality of ${\vec{x}}_{i}$ is equal to the dimensionality of its *l*-th nearest neighbor ${\vec{t}}_{il}\in T_{i}$; $T_{i}$ is a subset of the original dataset X containing nearest neighbors of the *i*-th object; and $d_{il}\in \mathbb{R}$ is the scalar distance value between the *i*-th object and its *l*-th nearest neighbor from the $T_{i}$ set.

As a result, for every object ${\vec{x}}_{i}\in X$ the dimensionality reduction algorithm determines a set $D_{i}=\left\{d_{i1},\ldots ,d_{il},\ldots ,d_{ik}\right\}$ containing the distances between ${\vec{x}}_{i}$ and each of its k nearest neighbors.

After computing the distances to each of the k nearest neighbors of ${\vec{x}}_{i}$, a fuzzy simplicial set is constructed, represented as a vector ${\vec{\mu }}_{i}\in {\mathbb{R}}^{n}$, where n denotes the object count in the original high dimensional dataset. In order to construct the ${\vec{\mu }}_{i}$ vector for every *i*-th object, the algorithm searches for $\rho_{i}\in D_{i}$, such that $\forall d_{il}\in D_{i}:\rho_{i}\leq d_{il}$. After that, a binary search is performed in order to find $\sigma_{i}$, which satisfies the following condition:

(2) $$\sum_{l=1}^{k}e^{\left(\frac{\rho_{i}-d_{il}}{\sigma_{i}}\right)}=\log_{2}k,\;$$

where i is the number of an object from the X set; l is the number of one of the nearest neighbors of the *i*-th object; k denotes nearest neighbor count; $\sigma_{i}\in \mathbb{R}$ is the target variable; $\rho_{i}\in D_{i}$ is the distance between the object ${\vec{x}}_{i}$ and its nearest neighbor from the $T_{i}$ set containing k neighbors; and $d_{il}\in D_{i}$ denotes the distance between the object ${\vec{x}}_{i}$ and its *l*-th neighbor from the $T_{i}$ set.

After determining $\rho_{i}$ and finding $\sigma_{i}$ satisfying (2) for every *i*-th object ${\vec{x}}_{i}$ from the original multidimensional dataset X, a sparse vector ${\vec{\mu }}_{i}\in {\mathbb{R}}^{n}$ is constructed. Every *j*-th scalar component of the ${\vec{\mu }}_{i}$ vector is represented by a fuzzy value indicating how similar the *i*-th and *j*-th objects from the X set are. Assuming i={1,2,…n} and j={1,2,…,n}, where n denotes object count in the multidimensional X set, if the two objects, ${\vec{x}}_{i}$ and ${\vec{x}}_{j}$, are not neighbors, then the *j*-th component $\mu_{ij}$ from the ${\vec{\mu }}_{i}$ vector is set to 0.

If the two objects, ${\vec{x}}_{i}$ and ${\vec{x}}_{j}$, are neighbors, then $\mu_{ij}$ is computed according to:

(3) $$\mu_{ij}=e^{\left(\frac{\rho_{i}-d_{ij}}{\sigma_{i}}\right)},\;$$

where i is the object number for which the ${\vec{\mu }}_{i}$ vector is being constructed; j is the number of a possible neighbor of the *i*-th object from the X set, and also the number of a component of the ${\vec{\mu }}_{i}$ vector, j={1,2,…,n}; $\rho_{i}$ is the minimum distance from the $D_{i}$ set; $d_{ij}$ is the distance between ${\vec{x}}_{i}$ and ${\vec{x}}_{j}$; and the dimensionality of the ${\vec{\mu }}_{i}$ vector is n, where n denotes object count in the multidimensional dataset X; $\mu_{ij}\in \left[0,1\right]$.

As a result, for every object ${\vec{x}}_{i}\in X$ a sparse vector ${\vec{\mu }}_{i}\in {\mathbb{R}}^{n}$ is obtained, which encodes fuzzy similarities between the *i*-th object and every *j*-th object belonging to the original high dimensional dataset X. Given that i={1,2,…n}, the algorithm constructs a sparse weighted adjacency matrix $M\in {\mathbb{R}}^{n\times n}$, where n rows are represented by n sparse fuzzy vectors ${\vec{\mu }}_{i}$. The weighted adjacency matrix M represents a fuzzy weighted oriented graph encoding pairwise similarities of objects from X, M is not symmetric.

On the next step, the asymmetric matrix M is symmetrized using probabilistic t-conorm according to the following formula:

(4) $$\mu_{ij}\leftarrow \mu_{ij}+\mu_{ji}-\mu_{ij}\mu_{ji},$$

where i and j are numbers of rows and columns in the M matrix, respectively, noting that $\mu_{ii}$ and $\mu_{jj}$ are equal to 0. As a result, the adjacency matrix M becomes symmetric.

### 2.2. Loss Function Optimization in the UMAP Algorithm

The initial low dimensional representations of high dimensional objects given by *h*-dimensional vectors from the X set in the ${\mathbb{R}}^{m}$ space are computed using spectral embedding [8], assuming m≤h. After applying spectral embedding to the X set, the matrix $Y\in {\mathbb{R}}^{n\times m}$ is obtained, where n denotes object count in the original dataset X, and m denotes the dimensionality of the target low dimensional space. After computing the initial locations of objects from X in the ${\mathbb{R}}^{m}$ space, the algorithm starts the loss function optimization process. According to [22], the original UMAP algorithm implementation uses weighted fuzzy cross entropy with reduced repulsion as the loss function:

(5) $$L_{1}\left(M,Y\right)=\sum_{i=1}^{n}\sum_{j=1}^{n}\left(\mu_{ij}\ln\frac{\mu_{ij}}{\nu_{ij}}+\frac{\sum_{k=1}^{n}\mu_{ik}}{2n}\ln\left(\frac{1-\mu_{ij}}{1-\nu_{ij}}\right)\right),$$

where $M\in {\mathbb{R}}^{n\times n}$ denotes the symmetric adjacency matrix, containing fuzzy values, encoding pairwise similarities of high dimensional objects from the X set (see Section 2.1); $Y\in {\mathbb{R}}^{n\times m}$ denotes representations of n objects in the low dimensional space ${\mathbb{R}}^{m}$; $\mu_{ij}\in \left[0,\;1\right]$ denotes a scalar value representing fuzzy similarity of *i*-th and *j*-th high dimensional objects from the original X set; and $\nu_{ij}\in \left[0,\;1\right]$ denotes a scalar value representing fuzzy similarity of *i*-th and *j*-th objects in low dimensional space ${\mathbb{R}}^{m}$.

In order to determine the pairwise similarity $\nu_{ij}$ of *i*-th and *j*-th objects represented by *i*-th and *j*-th rows of the $Y\in {\mathbb{R}}^{n\times m}$ matrix in the low dimensional space ${\mathbb{R}}^{m}$ the following formula is used:

(6) $$\nu_{ij}={\left(1+ad_{ij}^{2b}\right)}^{-1},$$

where $d_{ij}$ denotes the scalar distance value between the *i*-th and *j*-th objects, ${\vec{y}}_{i}$ and ${\vec{y}}_{j}$, represented by rows in the Y matrix, the $d_{ij}$ value can be computed using the Euclidean distance Formula (1), assuming ${\vec{x}}_{i}$ and ${\vec{t}}_{il}$ vectors in (1) are replaced with ${\vec{y}}_{i}$ and ${\vec{y}}_{j}$ respectively, and h is replaced with m in (1); a and b are the coefficients that are chosen by non-linear least squares fitting of (6) against the following curve:

(7) $$\psi_{ij}=\{\begin{array}{rr} 1, & \;\;d_{ij}\leq d_{min}\\ e^{\left(d_{min}-d_{ij}\right)}, & \;\;d_{ij}>d_{min} \end{array},$$

where $d_{ij}$ denotes the scalar distance value between the *i*-th and *j*-th objects, ${\vec{y}}_{i}$ and ${\vec{y}}_{j}$, represented by rows in the Y matrix, $d_{min}$ is the hyperparameter of the UMAP algorithm, the recommended values of $d_{min}$ belong to (0,1] and affect the density of the clusters formed during the loss function (5) optimization process in the low dimensional space ${\mathbb{R}}^{m}$ by the objects contained in the Y matrix.

In the UMAP algorithm, the optimization of the loss (5) is performed using stochastic gradient descent [10]. The locations of objects that are represented by rows in the matrix $Y\in {\mathbb{R}}^{n\times m}$ are modified on every iteration of the stochastic gradient descent algorithm in order to minimize the loss function.

Stochastic gradient descent is a first-order optimization method that exploits the information on values and gradients of a function being optimized. In order to apply a gradient-based algorithm, the gradients of a loss function have to be determined either analytically or numerically. In this paper, we analytically derive the gradients of all of the considered loss functions, this allows us to save the computational time required to determine the gradients numerically.

In order to derive the gradients, the loss (5) can be transformed into:

(8) $$L_{1}\left(M,Y\right)=\sum_{i=1}^{n}\sum_{j=1}^{n}\left(\mu_{ij}\ln\mu_{ij}-\mu_{ij}\ln\nu_{ij}+\frac{\sum_{k=1}^{n}\mu_{ik}}{2n}\ln\left(1-\mu_{ij}\right)-\frac{\sum_{k=1}^{n}\mu_{ik}}{2n}\ln\left(1-\nu_{ij}\right)\right),$$

The terms that do not depend on the Y matrix in Equation (8) are constant on every iteration of the optimization algorithm. After removing the constant terms and replacing $\nu_{ij}$ according to (6), Equation (8) is transformed into the following shape:

(9) $$L_{1}^{\sim }\left(M,Y\right)=-\sum_{i=1}^{n}\sum_{j=1}^{n}\left(\mu_{ij}\ln\frac{1}{\left(1+ad_{ij}^{2b}\right)}+\frac{\sum_{k=1}^{n}\mu_{ik}}{2n}\ln\left(1-\frac{1}{\left(1+ad_{ij}^{2b}\right)}\right)\right),$$

After splitting the function (9) into attractive component $L_{a}^{\sim }$ and repulsive component $L_{b}^{\sim }$ that can be independently differentiated, we get the following equation:

(10) $$L_{1}^{\sim }=L_{a}^{\sim }+L_{b}^{\sim }=-\sum_{i=1}^{n}\sum_{j=1}^{n}\left(\mu_{ij}\ln\frac{1}{\left(1+ad_{ij}^{2b}\right)}\right)-\sum_{i=1}^{n}\sum_{j=1}^{n}\left(\frac{\sum_{k=1}^{n}\mu_{ik}}{2n}\ln\left(1-\frac{1}{\left(1+ad_{ij}^{2b}\right)}\right)\right),$$

The first order partial derivative of $L_{a}^{\sim }$ (10) with respect to $d_{ij}$ is given by:

(11) $$\begin{array}{ll} \frac{\delta L_{a}^{\sim }}{\delta d_{ij}}=-\overset{n}{\underset{i=1}{\sum }}\overset{n}{\underset{j=1}{\sum }} & \left(\mu_{ij}\ln\left(\frac{1}{1+ad_{ij}^{2b}}\right)\right)\frac{\delta }{\delta d_{ij}}\;\\ & =-\overset{n}{\underset{i=1}{\sum }}\overset{n}{\underset{j=1}{\sum }}\left(\mu_{ij}\left(1+ad_{ij}^{2b}\right)\left(\left(\frac{1}{1+ad_{ij}^{2b}}\right)\frac{\delta }{\delta d_{ij}}\right)\right)\\ & =-\overset{n}{\underset{i=1}{\sum }}\overset{n}{\underset{j=1}{\sum }}\left(\mu_{ij}\left(1+ad_{ij}^{2b}\right)\left(\frac{-1}{{\left(1+ad_{ij}^{2b}\right)}^{2}}\right)\left(\left(1+ad_{ij}^{2b}\right)\frac{\delta }{\delta d_{ij}}\right)\right)\\ & =\overset{n}{\underset{i=1}{\sum }}\overset{n}{\underset{j=1}{\sum }}\left(\mu_{ij}\left(\frac{2abd_{ij}^{2b-1}}{1+ad_{ij}^{2b}}\right)\right) \end{array}$$

The first order partial derivative of $L_{b}^{\sim }$ (10) with respect to $d_{ij}$ is given by:

(12) $$\begin{array}{ll} \frac{\delta L_{r}^{\sim }}{\delta d_{ij}}=-\overset{n}{\underset{i=1}{\sum }}\overset{n}{\underset{j=1}{\sum }} & \left(\frac{\sum_{k=1}^{n}\mu_{ik}}{2n}\ln\left(1-\frac{1}{1+ad_{ij}^{2b}}\right)\right)\frac{\delta }{\delta d_{ij}}\;\\ & =-\overset{n}{\underset{i=1}{\sum }}\overset{n}{\underset{j=1}{\sum }}\left(\frac{\sum_{k=1}^{n}\mu_{ik}}{2n}\left(\frac{1+ad_{ij}^{2b}}{ad_{ij}^{2b}}\right)\left(\left(1-\frac{1}{1+ad_{ij}^{2b}}\right)\frac{\delta }{\delta d_{ij}}\right)\right)\\ & =-\overset{n}{\underset{i=1}{\sum }}\overset{n}{\underset{j=1}{\sum }}\left(\frac{\sum_{k=1}^{n}\mu_{ik}}{2n}\left(\frac{1+ad_{ij}^{2b}}{ad_{ij}^{2b}}\right)\left(\frac{1}{{\left(1+ad_{ij}^{2b}\right)}^{2}}\right)\left(\left(1+ad_{ij}^{2b}\right)\frac{\delta }{\delta d_{ij}}\right)\right)\\ & =-\overset{n}{\underset{i=1}{\sum }}\overset{n}{\underset{j=1}{\sum }}\left(\frac{\sum_{k=1}^{n}\mu_{ik}}{2n}\left(\frac{2abd_{ij}^{2b-1}}{ad_{ij}^{2b}\left(1+ad_{ij}^{2b}\right)}\right)\right)\\ & =-\overset{n}{\underset{i=1}{\sum }}\overset{n}{\underset{j=1}{\sum }}\left(\frac{\sum_{k=1}^{n}\mu_{ik}}{2n}\left(\frac{2b}{d_{ij}\left(1+ad_{ij}^{2b}\right)}\right)\right). \end{array}$$

Hence, the first order partial derivative of $L_{1}^{\sim }$ with respect to $d_{ij}$ is given by:

(13) $$\frac{\delta L_{1}^{\sim }}{\delta d_{ij}}=\frac{\delta L_{a}^{\sim }}{\delta d_{ij}}+\frac{\delta L_{r}^{\sim }}{\delta d_{ij}}=\overset{n}{\underset{i=1}{\sum }}\overset{n}{\underset{j=1}{\sum }}\left(\mu_{ij}\left(\frac{2abd_{ij}^{2b-1}}{1+ad_{ij}^{2b}}\right)-\frac{\sum_{k=1}^{n}\mu_{ik}}{2n}\left(\frac{2b}{d_{ij}\left(1+ad_{ij}^{2b}\right)}\right)\right).\;$$

During the optimization process of the loss function (5) using the gradient (13) the original UMAP implementation also respects the derivative of the $d_{ij}$ Euclidean distance metric. UMAP uses a sampling-based approach, meaning that on every iteration of the original UMAP algorithm, the attractive force $L_{attr}^{UMAP}$ is applied to every pair of objects from the Y set in case the objects are neighbors, with probability determined by the fuzzy value $\mu_{ij}\in \left[0,\;1\right]$ indicating the similarity of the two objects. If the two objects are not nearest neighbors, then they are spread away from each other by applying repulsive force $L_{rep}^{UMAP}$ to the objects. The forces are given by [10]:

(14) $$L_{attr}^{UMAP}=\frac{-2abd_{ij}^{2\left(b-1\right)}}{\left(1+ad_{ij}^{2b}\right)}\left({\vec{y}}_{i}-{\vec{y}}_{j}\right),\;\;L_{rep}^{UMAP}=\frac{2b}{d_{ij}^{2}\left(1+ad_{ij}^{2b}\right)}\left({\vec{y}}_{i}-{\vec{y}}_{j}\right)\;$$

The signs of the forces in (14) differ from the signs of the terms in (13) due to the fact that during loss function minimization using gradient descent the algorithm is moving towards the negative gradient of the loss function.

### 2.3. Fuzzy Cross Entropy Loss

Other fuzzy information discrimination measures exist [20,21], except the weighted fuzzy cross entropy loss with reduced repulsion (5), that is optimized in the original UMAP implementation, using gradient descent with a sampling-based approach. In this study, we investigate the applicability of other information discrimination measures in the UMAP algorithm. One such measure is fuzzy cross entropy [20,21], the simplest measure of information discrimination between two fuzzy sets, this measure was derived from Shannon entropy [23].

Fuzzy cross entropy can be used in UMAP while estimating how similar high dimensional objects from X and their low dimensional representations given by rows in Y are. In UMAP, high dimensional objects are first transformed into a weighted adjacency matrix $M\in {\mathbb{R}}^{n\times n}$, the transformation process is described in Section 2.1. The initial low dimensional representations $Y\in {\mathbb{R}}^{n\times m}$ of objects from the X set are computed by applying spectral embedding [8] to X, assuming m is the dimensionality of the target low dimensional space. Similar to (5), fuzzy cross entropy used to measure information discrimination between the weighted adjacency matrix M and low dimensional representations Y is given by the following equation:

(15) $$L_{2}\left(M,Y\right)=\sum_{i=1}^{n}\sum_{j=1}^{n}\left(\mu_{ij}\ln\frac{\mu_{ij}}{\nu_{ij}}+\left(1-\mu_{ij}\right)\ln\left(\frac{1-\mu_{ij}}{1-\nu_{ij}}\right)\right),$$

where $M\in {\mathbb{R}}^{n\times n}$ denotes the symmetric weighted adjacency matrix, where every *i*-th row represents the *i*-th object from the X set and contains fuzzy values describing how similar the *i*-th object is to every other object from the X set; n denotes object count in the original dataset X; $Y\in {\mathbb{R}}^{n\times m}$ denotes low dimensional representation of n objects from X; m denotes the dimensionality of the target low dimensional space; $\mu_{ij}\in M$ denotes the fuzzy value describing the similarity of the *i*-th and *j*-th objects in high dimensional space X; and $\nu_{ij}$ denotes the fuzzy value describing the similarity of the *i*-th and *j*-th objects in low dimensional space ${\mathbb{R}}^{m}$, $\nu_{ij}$ value is computed according to (6).

Similar to (5) and (8), Equation (5) can be transformed using the properties of the logarithmic functions, and the constants that do not depend on Y can be ignored during the optimization process. Similar to (9), replacing $\nu_{ij}$ according to (6) transforms (15) into the following equation:

(16) $$L_{2}^{\sim }\left(M,Y\right)=-\sum_{i=1}^{n}\sum_{j=1}^{n}\left(\mu_{ij}\ln\frac{1}{\left(1+ad_{ij}^{2b}\right)}+\left(1-\mu_{ij}\right)\ln\left(1-\frac{1}{\left(1+ad_{ij}^{2b}\right)}\right)\right),$$

where a and b denote the coefficients selected before the optimization process starts by non-linear least squares fitting of (6) against the curve (7), and $d_{ij}$ denotes the distance between *i*-th and *j*-th objects in the low dimensional space ${\mathbb{R}}^{m}$.

While the only difference between (9) and (16) is in the repulsive component weight, the first-order partial derivative of (16) with respect to $d_{ij}$, similar to (13), is given by:

(17) $$\frac{\delta L_{2}^{\sim }}{\delta d_{ij}}=\sum_{i=1}^{n}\sum_{j=1}^{n}\left(\mu_{ij}\left(\frac{2abd_{ij}^{2b-1}}{1+ad_{ij}^{2b}}\right)-\left(1-\mu_{ij}\right)\left(\frac{2b}{d_{ij}\left(1+ad_{ij}^{2b}\right)}\right)\right),$$

### 2.4. Symmetric Fuzzy Cross Entropy Loss

Symmetric fuzzy cross entropy [20,21] is a symmetric modification of (15) and can also be used to quantify the similarity of the graph $M\in {\mathbb{R}}^{n\times n}$ and the matrix Y containing n objects belonging to the low dimensional space ${\mathbb{R}}^{m}$. Similar to (15), in the considered problem, symmetric fuzzy cross entropy is given by:

(18) $$L_{3}\left(M,Y\right)=\sum_{i=1}^{n}\sum_{j=1}^{n}\left(\left(\mu_{ij}-\nu_{ij}\right)\ln\left(\frac{\mu_{ij}\left(1-\nu_{ij}\right)}{\nu_{ij}\left(1-\mu_{ij}\right)}\right)\right),$$

where $M\in {\mathbb{R}}^{n\times n}$ denotes the symmetric weighted adjacency matrix, where every *i*-th row represents the *i*-th object from the X set and contains fuzzy values $\mu_{ij}$ describing how similar the *i*-th object is to every other *j*-th object from the X set; n denotes object count in X; $Y\in {\mathbb{R}}^{n\times m}$ denotes the low dimensional representation of n objects from X; and $\nu_{ij}$ denotes a fuzzy value representing *i*-th and *j*-th object similarities in ${\mathbb{R}}^{m}$.

After the replacement of $\nu_{ij}$ in (18) according to (6), the transformation of (18) using the properties of the logarithmic functions gives the loss the following shape:

(19) $$\begin{array}{ll} L_{3}\left(M,Y\right)=\overset{n}{\underset{i=1}{\sum }}\overset{n}{\underset{j=1}{\sum }} & \left(\left(\mu_{ij}-\frac{1}{1+ad_{ij}^{2b}}\right)\ln\left(\frac{\mu_{ij}\left(1-\frac{1}{1+ad_{ij}^{2b}}\right)}{\frac{1}{1+ad_{ij}^{2b}}\left(1-\mu_{ij}\right)}\right)\right)\\ & =\overset{n}{\underset{i=1}{\sum }}\overset{n}{\underset{j=1}{\sum }}\left(\left(\mu_{ij}-\frac{1}{1+ad_{ij}^{2b}}\right)\ln\left(\frac{\mu_{ij}ad_{ij}^{2b}}{1-\mu_{ij}}\right)\right)\\ & =\overset{n}{\underset{i=1}{\sum }}\overset{n}{\underset{j=1}{\sum }}\left(\mu_{ij}\ln\left(\mu_{ij}ad_{ij}^{2b}\right)-\mu_{ij}\ln\left(1-\mu_{ij}\right)-\frac{\ln\left(\mu_{ij}ad_{ij}^{2b}\right)}{1+ad_{ij}^{2b}}+\frac{\ln\left(1-\mu_{ij}\right)}{1+ad_{ij}^{2b}}\right). \end{array}$$

After excluding terms that do not depend on $d_{ij}$, Equation (19) transforms into:

(20) $$L_{3}^{\sim }\left(M,Y\right)=\overset{n}{\underset{i=1}{\sum }}\overset{n}{\underset{j=1}{\sum }}\left(\mu_{ij}\ln\left(\mu_{ij}ad_{ij}^{2b}\right)-\frac{\ln\left(\mu_{ij}ad_{ij}^{2b}\right)}{1+ad_{ij}^{2b}}+\frac{\ln\left(1-\mu_{ij}\right)}{1+ad_{ij}^{2b}}\right).$$

The obtained function (20) can be then split into three terms $L_{a}^{\sim }$, $L_{b}^{\sim }$, and $L_{c}^{\sim }$:

(21) $$L_{3}^{\sim }=\overset{n}{\underset{i=1}{\sum }}\overset{n}{\underset{j=1}{\sum }}\left(\mu_{ij}\ln\left(\mu_{ij}ad_{ij}^{2b}\right)\right)-\overset{n}{\underset{i=1}{\sum }}\overset{n}{\underset{j=1}{\sum }}\left(\frac{\ln\left(\mu_{ij}ad_{ij}^{2b}\right)}{1+ad_{ij}^{2b}}\right)+\overset{n}{\underset{i=1}{\sum }}\overset{n}{\underset{j=1}{\sum }}\left(\frac{\ln\left(1-\mu_{ij}\right)}{1+ad_{ij}^{2b}}\right).$$

The first order partial derivative of $L_{a}^{\sim }$ with respect to $d_{ij}$ is given by:

(22) $$\frac{\delta L_{a}^{\sim }}{\delta d_{ij}}=\overset{n}{\underset{i=1}{\sum }}\overset{n}{\underset{j=1}{\sum }}\left(\mu_{ij}\ln\left(\mu_{ij}ad_{ij}^{2b}\right)\right)\frac{\delta }{\delta d_{ij}}=\overset{n}{\underset{i=1}{\sum }}\overset{n}{\underset{j=1}{\sum }}\left(2b\mu_{ij}d_{ij}^{-1}\right).$$

The first order partial derivative of $L_{b}^{\sim }$ with respect to $d_{ij}$ is given by:

(23) $$\begin{array}{ll} \frac{\delta L_{b}^{\sim }}{\delta d_{ij}}=-\overset{n}{\underset{i=1}{\sum }}\overset{n}{\underset{j=1}{\sum }} & \left(\frac{\ln\left(\mu_{ij}ad_{ij}^{2b}\right)}{1+ad_{ij}^{2b}}\right)\frac{\delta }{\delta d_{ij}}\\ & =\overset{n}{\underset{i=1}{\sum }}\overset{n}{\underset{j=1}{\sum }}\left(-\frac{2b\mu_{ij}ad_{ij}^{2b-1}}{\mu_{ij}ad_{ij}^{2b}\left(1+ad_{ij}^{2b}\right)}-\frac{-2abd_{ij}^{2b}\ln\left(\mu_{ij}ad_{ij}^{2b}\right)}{d_{ij}{\left(1+ad_{ij}^{2b}\right)}^{2}}\right)\\ & =\overset{n}{\underset{i=1}{\sum }}\overset{n}{\underset{j=1}{\sum }}\left(\frac{2b\left(-ad_{ij}^{2b}-1+ad_{ij}^{2b}\ln\left(\mu_{ij}ad_{ij}^{2b}\right)\right)}{d_{ij}{\left(1+ad_{ij}^{2b}\right)}^{2}}\right). \end{array}$$

The $L_{c}^{\sim }$ term of (21) can be differentiated trivially:

(24) $$\frac{\delta L_{c}^{\sim }}{\delta d_{ij}}=\overset{n}{\underset{i=1}{\sum }}\overset{n}{\underset{j=1}{\sum }}\left(\frac{\ln\left(1-\mu_{ij}\right)}{1+ad_{ij}^{2b}}\right)\frac{\delta }{\delta d_{ij}}=\overset{n}{\underset{i=1}{\sum }}\overset{n}{\underset{j=1}{\sum }}\left(-\frac{2bad_{ij}^{2b}\ln\left(1-\mu_{ij}\right)}{d_{ij}{\left(1+ad_{ij}^{2b}\right)}^{2}}\right).$$

The summation of the obtained derivatives (22), (23), and (24), leads to the following form of the derivative of (21) after several polynomial transformations:

(25) $$\frac{\delta L_{3}^{\sim }}{\delta d_{ij}}=\overset{n}{\underset{i=1}{\sum }}\overset{n}{\underset{j=1}{\sum }}\left(\frac{2b\left(\left(ad_{ij}^{2b}+1\right)\left(a\mu_{ij}d_{ij}^{2b}+\mu_{ij}-1\right)-ad_{ij}^{2b}\ln\left(1-\mu_{ij}\right)+ad_{ij}^{2b}\ln\left(\mu_{ij}ad_{ij}^{2b}\right)\right)}{d_{ij}{\left(1+ad_{ij}^{2b}\right)}^{2}}\right).$$

### 2.5. Modified Fuzzy Cross Entropy Loss

The modified fuzzy cross entropy measure of information discrimination between two sets was proposed in [23]. Modified fuzzy cross entropy is an asymmetric measure. Similar to the considered losses (5), (15), and (18), the modified fuzzy cross entropy loss applied to low dimensional embedding construction in UMAP is given by:

(26) $$L_{4}\left(M,Y\right)=\overset{n}{\underset{i=1}{\sum }}\overset{n}{\underset{j=1}{\sum }}\left(\mu_{ij}\ln\frac{\mu_{ij}}{\frac{1}{2}\mu_{ij}+\frac{1}{2}\nu_{ij}}+\left(1-\mu_{ij}\right)\ln\left(\frac{1-\mu_{ij}}{1-\frac{1}{2}\left(\mu_{ij}+\nu_{ij}\right)}\right)\right),\;$$

where $M\in {\mathbb{R}}^{n\times n}$ denotes the symmetric weighted adjacency matrix, every *i*-th row of M represents the *i*-th object from the X set and contains fuzzy values $\mu_{ij}$ describing how similar the *i*-th object is to every other *j*-th object from the X set; n denotes object count in X; $Y\in {\mathbb{R}}^{n\times m}$ denotes the low dimensional representation of n objects from X; and $\nu_{ij}$ denotes a fuzzy value representing *i*-th and *j*-th object similarities in ${\mathbb{R}}^{m}$.

The transformation of (26) in a fashion similar to (5), (15), and (18), by using the properties of logarithmic functions and removing the constant terms, leads to the following:

(27) $$L_{4}^{\sim }\left(M,Y\right)=-\overset{n}{\underset{i=1}{\sum }}\overset{n}{\underset{j=1}{\sum }}\left(\mu_{ij}\ln\left(\frac{1}{2}\mu_{ij}+\frac{1}{2}\nu_{ij}\right)+\left(1-\mu_{ij}\right)\ln\left(1-\frac{1}{2}\left(\mu_{ij}+\nu_{ij}\right)\right)\right),$$

After replacing $\nu_{ij}$ with (6) and splitting (27) into two terms, (27) transforms into:

(28) $$L_{4}^{\sim }\left(M,Y\right)=-\overset{n}{\underset{i=1}{\sum }}\overset{n}{\underset{j=1}{\sum }}\left(\mu_{ij}\ln\left(\frac{1}{2}\mu_{ij}+\frac{1}{2\left(1+ad_{ij}^{2b}\right)}\right)\right)-\overset{n}{\underset{i=1}{\sum }}\overset{n}{\underset{j=1}{\sum }}\left(\left(1-\mu_{ij}\right)\ln\left(1-\frac{1}{2}\mu_{ij}-\frac{1}{2\left(1+ad_{ij}^{2b}\right)}\right)\right),$$

First-order partial derivative of the first term $L_{a}^{\sim }$ in (28) with respect to $d_{ij}$ is given by:

(29) $$\begin{array}{ll} \frac{\delta L_{a}^{\sim }}{\delta d_{ij}}=-\overset{n}{\underset{i=1}{\sum }}\overset{n}{\underset{j=1}{\sum }} & \left(\mu_{ij}\ln\left(\frac{1}{2}\mu_{ij}+\frac{1}{2}{\left(1+ad_{ij}^{2b}\right)}^{-1}\right)\right)\frac{\delta }{\delta d_{ij}}\\ & =-\overset{n}{\underset{i=1}{\sum }}\overset{n}{\underset{j=1}{\sum }}\left(\mu_{ij}{\left(\frac{1}{2}\mu_{ij}+\frac{1}{2+2ad_{ij}^{2b}}\right)}^{-1}\left(\left(\frac{1}{2}{\left(1+ad_{ij}^{2b}\right)}^{-1}\right)\frac{\delta }{\delta d_{ij}}\right)\right)\\ & =\overset{n}{\underset{i=1}{\sum }}\overset{n}{\underset{j=1}{\sum }}\left(\left(\frac{\mu_{ij}\left(1+ad_{ij}^{2b}\right)}{\left(\mu_{ij}+\mu_{ij}ad_{ij}^{2b}+1\right)}\right)\left(\frac{2abd_{ij}^{2b-1}}{{\left(1+ad_{ij}^{2b}\right)}^{2}}\right)\right)\\ & =\overset{n}{\underset{i=1}{\sum }}\overset{n}{\underset{j=1}{\sum }}\left(\frac{2\mu_{ij}abd_{ij}^{2b-1}}{\left(\mu_{ij}+\mu_{ij}ad_{ij}^{2b}+1\right)\left(1+ad_{ij}^{2b}\right)}\right). \end{array}$$

First-order partial derivative of the second term $L_{b}^{\sim }$ in (28) with respect to $d_{ij}$ is:

(30) $$\begin{array}{cc} \frac{\delta L_{r}^{\sim }}{\delta d_{ij}}=-\overset{n}{\underset{i=1}{\sum }}\overset{n}{\underset{j=1}{\sum }} & \left(\left(1-\mu_{ij}\right)\ln\left(1-\frac{1}{2}\mu_{ij}-\frac{1}{2}{\left(1+ad_{ij}^{2b}\right)}^{-1}\right)\right)\frac{\delta }{\delta d_{ij}}\\ & =-\overset{n}{\underset{i=1}{\sum }}\overset{n}{\underset{j=1}{\sum }}\left(\left(1-\mu_{ij}\right){\left(1-\frac{1}{2}\mu_{ij}-\frac{1}{2\left(1+ad_{ij}^{2b}\right)}\right)}^{-1}\left(\left(-\frac{1}{2}{\left(1+ad_{ij}^{2b}\right)}^{-1}\right)\frac{\delta }{\delta d_{ij}}\right)\right)\\ & =-\overset{n}{\underset{i=1}{\sum }}\overset{n}{\underset{j=1}{\sum }}\left(\left(\frac{\left(1-\mu_{ij}\right)\left(1+ad_{ij}^{2b}\right)}{2+2ad_{ij}^{2b}-\mu_{ij}-\mu_{ij}ad_{ij}^{2b}-1}\right)\left(\frac{1}{{\left(1+ad_{ij}^{2b}\right)}^{2}}\right)\left(2abd_{ij}^{2b-1}\right)\right)\\ & =\overset{n}{\underset{i=1}{\sum }}\overset{n}{\underset{j=1}{\sum }}\left(\frac{2\left(1-\mu_{ij}\right)abd_{ij}^{2b-1}}{\left(\mu_{ij}ad_{ij}^{2b}-2ad_{ij}^{2b}+\mu_{ij}-1\right)\left(1+ad_{ij}^{2b}\right)}\right). \end{array}$$

Hence, the first order partial derivative of (28) with respect to $d_{ij}$ is given by:

(31) $$\frac{\delta L_{4}^{\sim }}{\delta d_{ij}}=\overset{n}{\underset{i=1}{\sum }}\overset{n}{\underset{j=1}{\sum }}\left(\left(\frac{2abd_{ij}^{2b-1}}{1+ad_{ij}^{2b}}\right)\left(\left(\frac{\mu_{ij}}{\left(\mu_{ij}ad_{ij}^{2b}+\mu_{ij}+1\right)}\right)+\left(\frac{\left(1-\mu_{ij}\right)}{\left(\mu_{ij}ad_{ij}^{2b}-2ad_{ij}^{2b}+\mu_{ij}-1\right)}\right)\right)\right).$$

### 2.6. Adam Optimization Algorithm

First-order partial derivatives (13), (17), (25), (31) of the considered loss functions (5), (15), (18), (26) were obtained analytically. Hence, the locations of high dimensional objects from X in the low dimensional target space ${\mathbb{R}}^{m}$ can be optimized by applying first-order optimization methods to the discussed fuzzy losses. The Algorithm 1 [25] optimization algorithm is often used while training neural networks [27,28]. The pseudocode of the gradient-based Adam optimization algorithm is given by:
**Algorithm 1 Adam****Input**: $s_{0}$—initial solution, η, $\beta_{1}$, $\beta_{2}$—learning step sizes,  f1.set iteration number t to 02.initialize the $c_{0}$ and $v_{0}$ tensors filled with zeros3.set $\epsilon =10^{-8}$4.**while** the stop condition is not met **do**:5.t=t+16.$c_{t}=\beta_{1}\times c_{t-1}+\left(1-\beta_{1}\right)\times \nabla f\left(s_{t-1}\right)$7.$v_{t}=v_{t-1}-\left(1-\beta_{2}\right)\times \left(v_{t-1}-\nabla f^{2}\left(s_{t-1}\right)\right)$8.$s_{t}=\eta \times c_{t}\times {\left(\sqrt{v_{t}}+\epsilon \right)}^{-1}$9.**end loop**10.**return** $s_{t}$

The parameters of the Adam optimization algorithms $\beta_{1}$ and $\beta_{2}$ are often set to 0.9 and 0.999 respectively, the ϵ parameter is used to avoid division by zero, and the step size η is set depending on the considered domain. The dimensionality of the $c_{t}$ and $v_{t}$ vectors is equal to the dimensionality of the candidate solution $s_{0}$.

In the low dimensional embedding construction problem in UMAP, the Adam algorithm is applied to one of the considered loss functions. During the optimization process, the algorithm uses the weighted adjacency matrix $M\in {\mathbb{R}}^{n\times n}$ as the first argument in functions (5), (15), (18), (26) and n defines object count in the original high dimensional dataset X. The process of weighted adjacency matrix construction was described in Section 2.1. As the second argument in (5), (15), (18), (26), the algorithm uses the $Y\in {\mathbb{R}}^{n\times m}$ matrix, where m denotes the dimensionality of the target space.

Given that, the matrix Y is used as a candidate solution $s_{t}$ in Adam on every iteration t, the initial solution $s_{0}$ is constructed from the original high dimensional X set using spectral embedding [8]. The optimization process is stopped when the specified iteration limit is reached.

## 3. Numerical Experiment

### 3.1. Fuzzy Weighted Adjacency Matrix Construction

**Figure 1:**
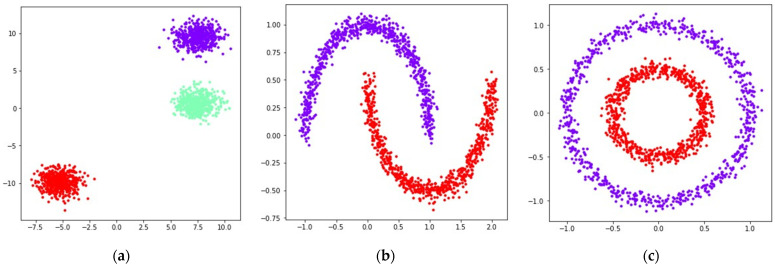
Locations of 1500 points belonging to the datasets generated by sklearn [29] in ${\mathbb{R}}^{2}$: (**a**) blobs; (**b**) moons, noise level is set to 0.05; (**c**) circles, noise level is set to 0.05, inner circle radius is equal to one half of the outer circle radius.

In order to compare the performance of the considered loss functions in the UMAP algorithm, we used datasets generated by the sklearn library [29]. The generated datasets contained 1500 points belonging to ${\mathbb{R}}^{2}$, separated into several noisy clusters of different shapes and sizes. Applying UMAP to datasets containing objects belonging to ${\mathbb{R}}^{2}$ allows one to get more context regarding the mutual displacement of objects in the original dataset, as the objects from ${\mathbb{R}}^{2}$ can be visualized as is. This allows one to compare the positions of objects from the original dataset with the positions of objects obtained after applying UMAP transformations using different loss functions. Visualizations of the original locations of the generated points in ${\mathbb{R}}^{2}$ are shown in Figure 1.

**Figure 2:**
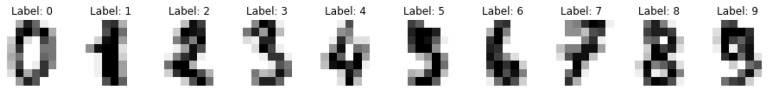
The visualization of 10 handwritten digits randomly chosen from the [30] dataset.

In addition, we considered the dataset [30] containing 1797 images of handwritten digits from zero to nine, the images were represented as matrices of shape ${\mathbb{R}}^{8\times 8}$. Every cell in such a matrix is characterized by color, encoded as an integer belonging to the [0, 16] interval. Every image from this dataset can be represented by a vector of shape ${\mathbb{R}}^{64}$, components of which are integers belonging to [0, 16]. The visualization of handwritten digits from the [30] dataset created with sklearn [29] is shown in Figure 2.

The UMAP algorithm was implemented in the Python programming language using such libraries as numpy [31] and numba [32], as described in Section 2.1. First, the UMAP algorithm searches for k nearest neighbors for every object in the original high dimensional dataset, and then computes distances to the k nearest neighbors. The k value is the hyperparameter of the UMAP algorithm. As we see later, choosing bigger k values might improve dataset global structure preservation while reducing the dimensionality. After finding the nearest neighbors and computing the distances to them, the $M\in {\mathbb{R}}^{n\times n}$ weighted adjacency matrix is built, representing a weighted unoriented graph, describing pairwise object similarities in the original dataset X, as described in Section 2.1.

**Figure 3:**
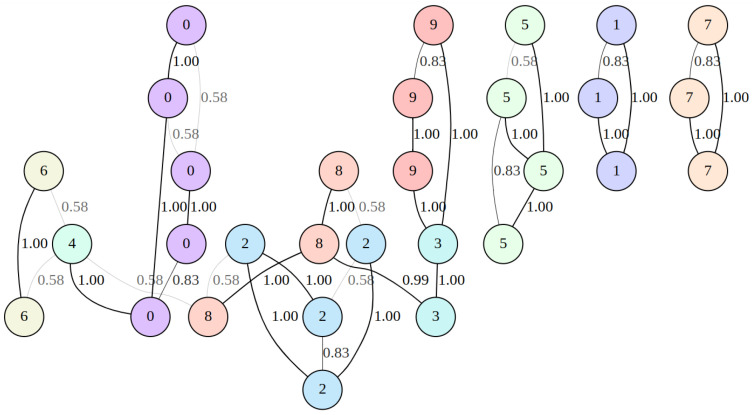
Graph represented by the weighted adjacency matrix $M\in {\mathbb{R}}^{30\times 30}$ that was built by the UMAP algorithm for 30 randomly chosen images from the [30] dataset with neighbors count k set to 2. The visualization was obtained using the graphviz tool [33].

For 30 randomly chosen hand-written digits from the dataset [30] with nearest neighbor count k set to two, the neighborhood graph was built by the UMAP algorithm. The graph was represented by a weighted adjacency matrix $M\in {\mathbb{R}}^{30\times 30}$, as shown in Figure 3.

For the datasets that were generated with the sklearn library and contain 1500 points belonging to the ${\mathbb{R}}^{2}$ space, UMAP computed distances to the k nearest neighbors, and constructed a weighted adjacency matrix $M\in {\mathbb{R}}^{1500\times 1500}$. For the dataset containing 1797 hand-written digits represented by 64-dimensional vectors, UMAP computed distances to the k nearest neighbors and constructed a weighted adjacency matrix $M\in {\mathbb{R}}^{1797\times 1797}$.

### 3.2. Coefficients Fitting

**Figure 4:**
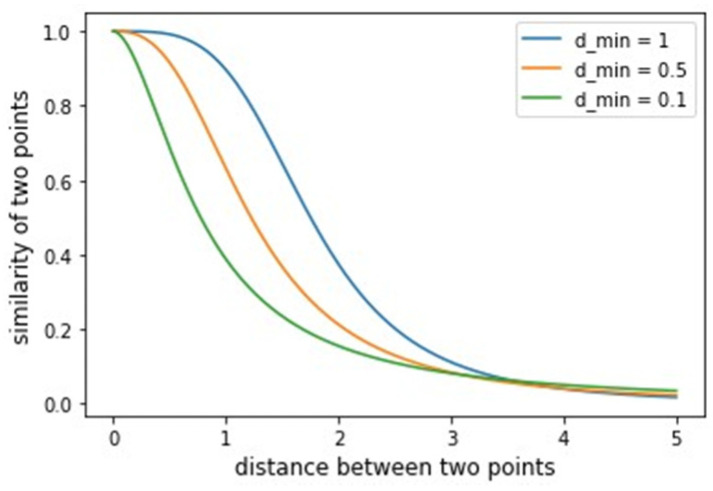
The dependency of curve (6) shape on the $d_{min}$ parameter value.

After constructing the fuzzy weighted undirected graph for each of the considered datasets, UMAP performs a search for a and b coefficients in function (6). The coefficients are chosen by least squares fitting of (6) against the curve (7). The shape of the curve (7) depends on the parameter $d_{min}$. The plot illustrating how the $d_{min}$ variable affects the curve (6) shape is shown in Figure 4.

The function (6) maps pairwise distances between two nearest neighbors into fuzzy values measuring the similarity of two objects $\nu_{ij}$. According to Figure 4, different $d_{min}$ parameter values lead to a curve different shape (6), meaning that different a and b coefficients get selected. With small values of $d_{min}$, clusters in UMAP become denser.

### 3.3. Weighted Fuzzy Cross Entropy Loss Optimization

Using the weighted adjacency matrices obtained for each of the considered datasets with nearest neighbor count set k set to 10, and the a and b coefficients selected by least squares fitting of (6) against (7) with $d_{min}=\left\{0.1,1\right\}$, the weighted fuzzy cross entropy with reduced repulsion (5) was minimized using the Adam gradient-based optimization algorithm. The first-order partial derivative of (5) with respect to pairwise distances $d_{ij}$ is given by (13), so the gradients were computed on every iteration according to:

(32) $$\frac{\delta L_{1}^{\sim }}{\delta {\vec{y}}_{i}}=\overset{n}{\underset{j=1}{\sum }}\left[\left(\mu_{ij}\left(\frac{2abd_{ij}^{2b-1}}{1+ad_{ij}^{2b}}\right)-\frac{\sum_{k=1}^{n}\mu_{ik}}{2n}\left(\frac{2b}{d_{ij}\left(1+ad_{ij}^{2b}\right)}\right)\right)\left({\vec{y}}_{i}-{\vec{y}}_{j}\right)\right].\;$$

where ${\vec{y}}_{i}$ and ${\vec{y}}_{j}$ denote the *i*-th and *j*-th ${\mathbb{R}}^{2}$ representations of objects from the original dataset X; $d_{ij}$ denotes the distance between ${\vec{y}}_{i}$ and ${\vec{y}}_{j}$ in the ${\mathbb{R}}^{2}$ space, computed according to (1) on every iteration of the Adam algorithm; $\mu_{ij}\in M$ denotes pairwise similarity of the original *i*-th and *j*-th objects from the X dataset; and a and b denote the coefficients chosen by least squares fitting of (6) against (7) with a specified $d_{min}$ value.

**Figure 5:**
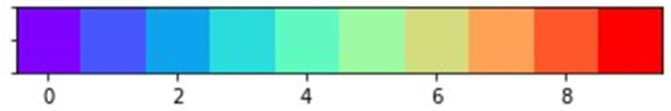
Colors and the corresponding digits.

The parameters of the Adam optimization algorithm are listed in Table 1. For the dataset containing hand-written digits, each digit was assigned with its own color. The colors and the corresponding digits are listed in Figure 5.

**Figure 6:**
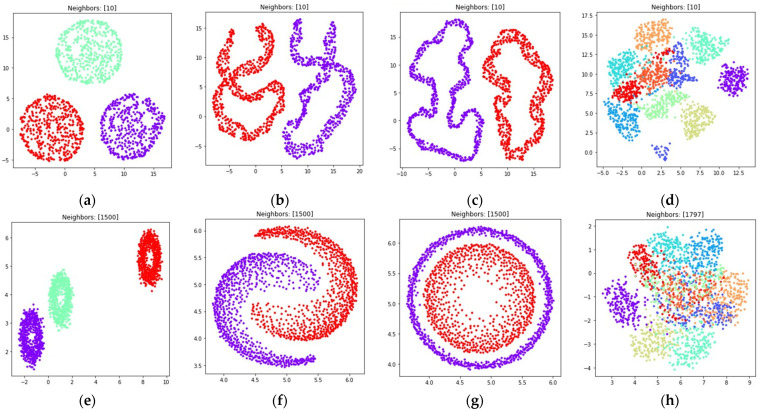
Locations of objects in ${\mathbb{R}}^{2}$ with dimensionality reduction performed by applying the UMAP algorithm, with low dimensional embedding optimized by the Adam algorithm and the gradient (32) with $d_{min}$ set to 1 for: (**a**) blobs, k=10; (**b**) moons, k=10; (**c**) circles, k=10; (**d**) handwritten digits, k=10; (**e**) moons, k=(n−1); (**f**) blobs, k=(n−1); (**g**) circles, k=(n−1); (**h**) handwritten digits, k=(n−1).

**Figure 7:**
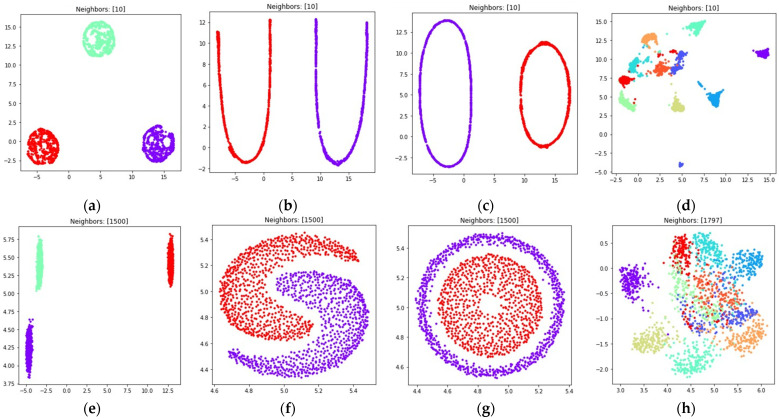
Locations of objects in ${\mathbb{R}}^{2}$ with dimensionality reduction performed by applying the UMAP algorithm, with low dimensional embedding optimized by the Adam algorithm and the gradient (32) with $d_{min}$ set to 0.1 for: (**a**) blobs, k=10; (**b**) moons, k=10; (**c**) circles, k=10; (**d**) handwritten digits, k=10; (**e**) moons, k=(n−1); (**f**) blobs, k=(n−1); (**g**) circles, k=(n−1); (**h**) handwritten digits, k=(n−1).

The obtained visualizations for all of the considered datasets are shown in Figure 6 and Figure 7. Figure 6 contains visualizations for $d_{min}=1$, Figure 7 contains visualizations for $d_{min}=0.1$. According to Figure 6 and Figure 7, the weighted fuzzy cross entropy measure with reduced repulsion the UMAP algorithm successfully separates objects into several clusters. With $d_{min}=1$, the clusters in ${\mathbb{R}}^{2}$ are less dense, compared to the clusters obtained with $d_{min}=0.1$.

According to Figure 6d,h and Figure 7d,h, the loss (5) works best when the nearest neighbor count k is set to a relatively small value. This happens due to the fact, that in this case the first term in (5) is equal to zero for all objects that are not nearest neighbors, as $\mu_{ij}=0$ for non-neighbors, as described in Section 2.1. On the one hand, this allows one to separate the objects into more dense clusters, by applying the attractive force only to the nearest neighbors on every iteration. On the other hand, with relatively small k values the information of the global structure of a high dimensional dataset might be lost. For example, the handwritten digits two and seven are similar, but their clusters, as shown in Figure 6d and Figure 7d, are separated from each other. The handwritten digits one and zero are less similar, however their clusters with $d_{min}$ set to one are rendered relatively close to each other.

With the sufficient increase of nearest neighbor count k by setting k=(n−1), where n denotes object count in the considered dataset, to preserve more of the global structure of high dimensional data, the algorithm sometimes struggles with local structure preservation, as shown in Figure 6h and Figure 7h. The first term in (32) stops being equal to zero for non-neighbors and the attractive force gets applied to every object in the dataset, but with different weighting terms $\mu_{ij}$.

### 3.4. Fuzzy Cross Entropy Loss Optimization

The fuzzy cross entropy loss is given by (15), and the first-order partial derivative of (15) with respect to $d_{ij}$ is given by (17). Using the obtained weighted adjacency matrices for the considered datasets with nearest neighbor count k set to 10 and (n−1), where n denotes object count in the original high dimensional dataset, and the a and b values in (6) obtained by nonlinear least squares fitting of (6) against (7) with $d_{min}\in \left\{0.1,1\right\}$, the fuzzy cross entropy loss was minimized using Adam. The parameters of the Adam algorithm are listed in Table 1. The gradient of fuzzy cross entropy (15) with derivative given by (17) was computed on every iteration of Adam according to:

(33) $$\frac{\delta L_{2}^{\sim }}{\delta {\vec{y}}_{i}}=\overset{n}{\underset{j=1}{\sum }}\left[\left(\mu_{ij}\left(\frac{2abd_{ij}^{2b-1}}{1+ad_{ij}^{2b}}\right)-\left(1-\mu_{ij}\right)\left(\frac{2b}{d_{ij}\left(1+ad_{ij}^{2b}\right)}\right)\right)\left({\vec{y}}_{i}-{\vec{y}}_{j}\right)\right].\;$$

where ${\vec{y}}_{i}$ and ${\vec{y}}_{j}$ denote the *i*-th and *j*-th ${\mathbb{R}}^{2}$ representations of objects from the original dataset X; $d_{ij}$ denotes the distance between ${\vec{y}}_{i}$ and ${\vec{y}}_{j}$ in the ${\mathbb{R}}^{2}$ space, computed according to (1) on every iteration of the Adam algorithm; $\mu_{ij}\in M$ denotes pairwise similarity of the original *i*-th and *j*-th objects from the X dataset; and a and b denote the learned coefficients in (6) for a particular $d_{min}$ value in (7).

**Figure 8:**
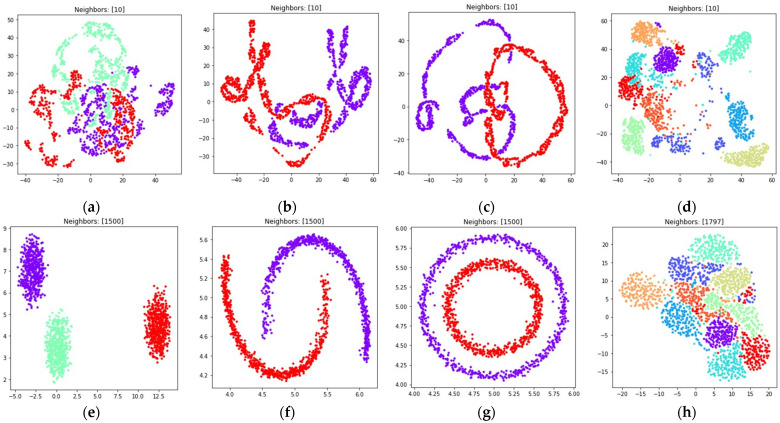
Locations of objects in ${\mathbb{R}}^{2}$ with dimensionality reduction performed by applying the UMAP algorithm, with low dimensional embedding optimized by the Adam algorithm, fuzzy cross entropy loss, and the gradient (33) with $d_{min}$ set to 1 for: (**a**) blobs, k=10; (**b**) moons, k=10; (**c**) circles, k=10; (**d**) handwritten digits, k=10; (**e**) moons, k=(n−1); (**f**) blobs, k=(n−1); (**g**) circles, k=(n−1); (**h**) handwritten digits, k=(n−1).

**Figure 9:**
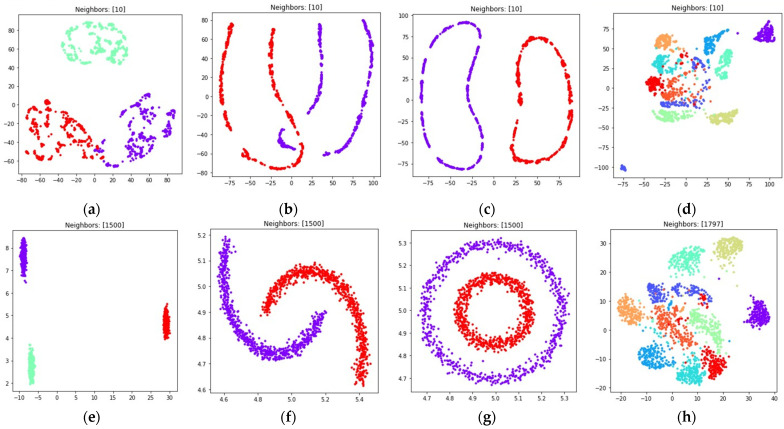
Locations of objects in ${\mathbb{R}}^{2}$ with dimensionality reduction performed by applying the UMAP algorithm, with low dimensional embedding optimized by the Adam algorithm, fuzzy cross entropy loss, and the gradient (33) with $d_{min}$ set to 0.1 for: (**a**) blobs, k=10; (**b**) moons, k=10; (**c**) circles, k=10; (**d**) handwritten digits, k=10; (**e**) moons, k=(n−1); (**f**) blobs, k=(n−1); (**g**) circles, k=(n−1); (**h**) handwritten digits, k=(n−1).

The visualizations of the considered datasets in the target low dimensional space ${\mathbb{R}}^{2}$ are shown in Figure 8 and Figure 9. The visualizations with $d_{min}$ set to 1 are shown in Figure 8, the visualizations with $d_{min}$ set to 0.1 are shown in Figure 9.

According to Figure 8 and Figure 9, with nearest neighbors count k set to (n−1), where n denotes object count in the original high dimensional dataset, the loss function (15) successfully separates objects into non-overlapping clusters. The global structure of the high dimensional datasets is preserved better when using (15) when compared to (5). According to the locations of clusters in Figure 8h and Figure 9h, the three and nine handwritten digits are similar, as well as two and seven, four and six, and their clusters are rendered close to each other. The zero and one digits are less similar, and their clusters are spread away from each other. According to Figure 8 and Figure 9, it is better to use the (15) loss with k=(n−1). With k=10 the algorithm might struggle to preserve global distances.

### 3.5. Symmetric Fuzzy Cross Entropy Loss Optimization

The symmetric fuzzy cross entropy is given by (18), and the first-order partial derivative of (18) with respect to $d_{ij}$ is given by (25). For the symmetric fuzzy cross entropy loss, the nearest neighbor count k was also set to 10 and (n−1), where n denotes object count. In the case of symmetric fuzzy cross entropy, we also expected that setting k=(n−1) would help to preserve the global structure of the data. The parameters of Adam were set according to Table 1, the gradient of (18) was computed based on its derivative (25) on every iteration according to the following formula:

(34) $$\frac{\delta L_{3}^{\sim }}{\delta {\vec{y}}_{i}}=\overset{n}{\underset{i=1}{\sum }}\left[\left(\frac{2b\left(\left(ad_{ij}^{2b}+1\right)\left(a\mu_{ij}d_{ij}^{2b}+\mu_{ij}-1\right)-ad_{ij}^{2b}\ln\left(1-\mu_{ij}\right)+ad_{ij}^{2b}\ln\left(\mu_{ij}ad_{ij}^{2b}\right)\right)}{d_{ij}{\left(1+ad_{ij}^{2b}\right)}^{2}}\right)\left({\vec{y}}_{i}-{\vec{y}}_{j}\right)\right].\;$$

where ${\vec{y}}_{i}$ and ${\vec{y}}_{j}$ denote the *i*-th and *j*-th ${\mathbb{R}}^{2}$ representations of objects from the original dataset X; $d_{ij}$ denotes the distance between ${\vec{y}}_{i}$ and ${\vec{y}}_{j}$ belonging to the ${\mathbb{R}}^{2}$ space computed according to (1) on every iteration of the Adam algorithm; $\mu_{ij}\in M$ denotes pairwise similarity of the original *i*-th and *j*-th objects from the X dataset; and a and b denote the learned coefficients in (6) for a particular $d_{min}$ value in (7).

**Figure 10:**
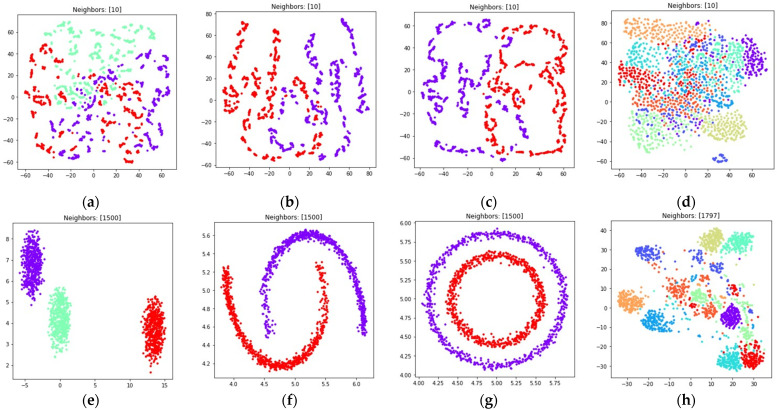
Locations of objects in ${\mathbb{R}}^{2}$ with dimensionality reduction performed by applying the UMAP algorithm, with low dimensional embedding optimized by the Adam algorithm, symmetric fuzzy cross entropy loss, and the gradient (34) with $d_{min}$ set to 1 for: (**a**) blobs, k=10; (**b**) moons, k=10; (**c**) circles, k=10; (**d**) handwritten digits, k=10; (**e**) moons, k=(n−1); (**f**) blobs, k=(n−1); (**g**) circles, k=(n−1); (**h**) handwritten digits, k=(n−1).

**Figure 11:**
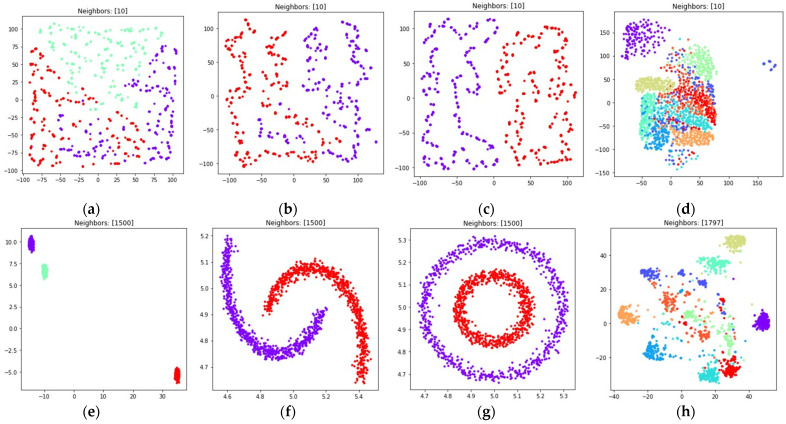
Locations of objects in ${\mathbb{R}}^{2}$ with dimensionality reduction performed by applying the UMAP algorithm, with low dimensional embedding optimized by the Adam algorithm, symmetric fuzzy cross entropy loss, and the gradient (34) with $d_{min}$ set to 0.1 for: (**a**) blobs, k=10; (**b**) moons, k=10; (**c**) circles, k=10; (**d**) handwritten digits, k=10; (**e**) moons, k=(n−1); (**f**) blobs, k=(n−1); (**g**) circles, k=(n−1); (**h**) handwritten digits, k=(n−1).

The obtained visualizations are shown in Figure 10 and Figure 11. According to the visualizations, the use of (18) with k=(n−1) also allows one to separate objects into dense clusters. The positions of the clusters shown in Figure 10 and Figure 11 are similar to the positions of clusters shown in Figure 8 and Figure 9. Larger k values lead to more dense and large clusters. Smaller k values lead to many small clusters, as shown in Figure 10a–c and Figure 11a–c.

### 3.6. Modified Fuzzy Cross Entropy Loss Optimization

The modified fuzzy cross entropy proposed in [23] is given by (26), the derivative of (26) is given by (31). The preliminary experiments have shown that with relatively small nearest neighbor k, the loss suffers with both local and global structure preservation of the original dataset. Hence, we set the k value to (n−1), where n denotes object count in X. The parameters of the Adam algorithm were set according to Table 1. The gradients of (26) were computed on every iteration according to the following formula:

(35) $$\frac{\delta L_{4}^{\sim }}{\delta {\vec{y}}_{i}}=\overset{n}{\underset{i=1}{\sum }}\overset{n}{\underset{j=1}{\sum }}\left[\left(\frac{2abd_{ij}^{2b-1}}{1+ad_{ij}^{2b}}\right)\left(\left(\frac{\mu_{ij}}{\left(\mu_{ij}ad_{ij}^{2b}+\mu_{ij}+1\right)}\right)+\left(\frac{\left(1-\mu_{ij}\right)}{\left(\mu_{ij}ad_{ij}^{2b}-2ad_{ij}^{2b}+\mu_{ij}-1\right)}\right)\right)\left({\vec{y}}_{i}-{\vec{y}}_{j}\right)\right].\;$$

where ${\vec{y}}_{i}$ and ${\vec{y}}_{j}$ denote the *i*-th and *j*-th ${\mathbb{R}}^{2}$ representations of objects from the original dataset X; $d_{ij}$ denotes the distance between ${\vec{y}}_{i}$ and ${\vec{y}}_{j}$ in the ${\mathbb{R}}^{2}$ space, computed according to (1) on every iteration of the Adam algorithm; $\mu_{ij}\in M$ denotes pairwise similarity of the original *i*-th and *j*-th objects from the X dataset; and a and b denote the coefficients chosen by least squares fitting of (6) against (7) with a specified $d_{min}$ value.

**Figure 12:**
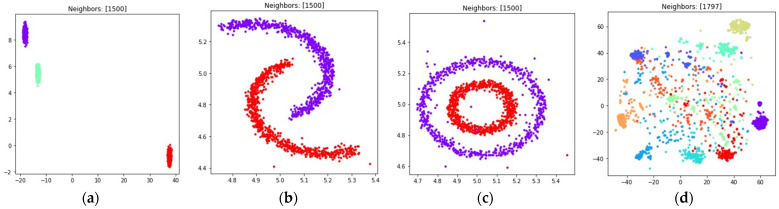
Locations of objects in ${\mathbb{R}}^{2}$ with dimensionality reduction performed by applying the UMAP algorithm, with low dimensional embedding optimized by the Adam algorithm, modified fuzzy cross entropy loss, and the gradient (35) with $d_{min}$ set to 0.1 for: (**a**) blobs, k=10; (**b**) moons, k=(n−1); (**c**) circles, k=(n−1); (**d**) handwritten digits, k=(n−1).

The obtained visualizations with $d_{min}=0.1$ are shown in Figure 12.

According to Figure 12, the modified fuzzy cross entropy that is given by (26) is also able to find clusters in high dimensional space and embed the clusters into ${\mathbb{R}}^{2}$. The locations and shapes of the clusters are similar to the locations obtained by using other losses, as shown in Figure 8, Figure 9, Figure 10 and Figure 11. However, as we see in Figure 12c,d, there is plenty of objects which do not belong to any of the clusters. The loss (26) did not manage to discover the clusters of handwritten digits such as five and eight.

## 4. Discussion

In this research, we considered different loss functions used during the low dimensional embedding construction process in the UMAP algorithm applied to multidimensional data visualization. In order to achieve this, we reimplemented the UMAP algorithm from scratch [10], with an intention to make the incorporation of custom losses into the original algorithm possible. The original implementation of the considered dimensionality reduction technique uses a sampling-based approach inspired with stochastic gradient descent while performing loss function optimization, and this leads to a different weighting of fuzzy cross entropy terms [22] when compared to traditional fuzzy cross entropy defined in [20,21]. Based on the findings published in [22], we explicitly defined the fuzzy cross entropy loss with reduced repulsion weight, derived the gradients analytically ignoring the normalization, and optimized the obtained loss using the first-order gradient-based Adam algorithm, without using the sampling-based approach. Other considered loss functions include the original fuzzy cross entropy without term weighting [20,21], symmetric fuzzy cross entropy [20,21], and modified fuzzy cross entropy, proposed in [23]. The gradients for all of the considered losses were determined analytically in order to make optimization possible using the first-order Adam algorithm without the need for numerical gradient computation.

During the numerical experiment, we considered both multidimensional and two-dimensional datasets. Mutual displacements of objects belonging to ${\mathbb{R}}^{2}$ can be easily visualized (see Figure 1), and then their positions can be compared with the embeddings obtained after applying UMAP-based transformations (see Figure 6, Figure 7, Figure 8, Figure 9, Figure 10, Figure 11 and Figure 12). This allows one to visually determine how good a manifold learning algorithm is at preserving the local and global structure of the original dataset when performing dimensionality reduction.

The obtained visualizations confirm that the fuzzy cross entropy loss with or without reduced repulsion, as well as the symmetric fuzzy cross entropy loss, is able to discover clusters in the original datasets and map them into the target space, preserving the structure of the original datasets. The visualizations of embeddings obtained by applying UMAP to datasets containing objects belonging to ${\mathbb{R}}^{2}$ show that the choice of a loss function greatly affects the result. The fuzzy cross entropy with reduced repulsion that is used in the original UMAP algorithm [22] works best with small nearest neighbor count values k, and is very good at preserving local structure (see Figure 6a–c). Other considered losses perform best with sufficiently large k values. For example, when k is set to (n−1), where n denotes object count in the original dataset, the algorithm preserves most of the global structure (see Figure 8 and Figure 10).

The visualizations of high dimensional handwritten digits show that the weighted fuzzy cross entropy loss with reduced repulsion is able to separate data into non-overlapping clusters only for relatively small neighbor counts k. With sufficiently large k values UMAP struggles to preserve local structure of the original high dimensional dataset (see Figure 6h and Figure 7h). Losses such as fuzzy cross entropy and symmetric fuzzy cross entropy with nearest neighbor count k set to (n−1), where n denotes object count in the original dataset, successfully preserve both the local and global structure of the original datasets. With k=(n−1), the symmetric fuzzy cross entropy loss (18) produces clusters with objects packed more densely, as shown in Figure 10h and Figure 11h, and the fuzzy cross entropy loss (15) distributes objects more uniformly in ${\mathbb{R}}^{2}$, while preserving the shape and mutual arrangement of the clusters, as shown in Figure 8h and Figure 9h. The use of the modified fuzzy cross entropy (26) leads to the inability of the algorithm to visualize non-overlapping clusters of some types of objects, as shown in Figure 12d.

## 5. Conclusions

The obtained results show that the use of fuzzy cross entropy without reduced repulsive weight, as well as symmetric cross entropy with sufficiently large nearest neighbor count k, can enhance the global structure preservation of the original dataset. This could be useful for the visual interpretation of high dimensional data in many different domains, such as medical diagnosis [34] or single cell RNA sequences clustering [35]. Dimensionality reduction algorithms also find their applications in data preprocessing [36] in order to enhance clustering or classification algorithm accuracy.

Further research could cover performance investigation of other fuzzy cross entropies used as loss functions in the UMAP algorithm, such as Tsallis divergence [37,38], fuzzy exponential cross entropy [39] and other divergence measures between two fuzzy sets. Additionally, further work could focus on deriving losses based on the principles highlighted in [19]. The approach to multidimensional data visualization presented in this paper, however, is not sampling-based, so further research could focus on developing sampling-based iterative schemes for the considered losses, similar to the scheme used in the UMAP reference implementation [10], aimed to improve the speed and reduce the computational complexity of the iterative loss function optimization process.

## Figures and Tables

**Table 1:** Parameters of the Adam optimization algorithm.

η	$\beta_{1}$	$\beta_{2}$	Iteration Limit
1	0.9	0.999	150

## Data Availability

Not applicable.
